# Use of Deep-Learning Genomics to Discriminate Healthy Individuals from Those with Alzheimer's Disease or Mild Cognitive Impairment

**DOI:** 10.1155/2021/3359103

**Published:** 2021-07-14

**Authors:** Lanlan Li, Yeying Yang, Qi Zhang, Jiao Wang, Jiehui Jiang, Alzheimer's Disease Neuroimaging Initiative

**Affiliations:** ^1^Institute of Biomedical Engineering, School of Communication and Information Engineering, Shanghai University, Shanghai 200444, China; ^2^LongHua Hospital, Shanghai University of Traditional Chinese Medicine, Shanghai 200032, China; ^3^School of Life Science, Shanghai University, Shanghai 200444, China; ^4^Indiana University School of Medicine, Indianapolis, IN 46202, USA

## Abstract

**Objectives:**

Alzheimer's disease (AD) is the most prevalent neurodegenerative disorder and the most common form of dementia in the elderly. Certain genes have been identified as important clinical risk factors for AD, and technological advances in genomic research, such as genome-wide association studies (GWAS), allow for analysis of polymorphisms and have been widely applied to studies of AD. However, shortcomings of GWAS include sensitivity to sample size and hereditary deletions, which result in low classification and predictive accuracy. Therefore, this paper proposes a novel deep-learning genomics approach and applies it to multitasking classification of AD progression, with the goal of identifying novel genetic biomarkers overlooked by traditional GWAS analysis.

**Methods:**

In this study, we selected genotype data from 1461 subjects enrolled in the Alzheimer's Disease Neuroimaging Initiative, including 622 AD, 473 mild cognitive impairment (MCI), and 366 healthy control (HC) subjects. The proposed deep-learning genomics (DLG) approach consists of three steps: quality control, coding of single-nucleotide polymorphisms, and classification. The ResNet framework was used for the DLG model, and the results were compared with classifications by simple convolutional neural network structure. All data were randomly assigned to one training/validation group and one test group at a ratio of 9 : 1. And fivefold cross-validation was used.

**Results:**

We compared classification results from the DLG model to those from traditional GWAS analysis among the three groups. For the AD and HC groups, the accuracy, sensitivity, and specificity of classification were, respectively, 98.78 ± 1.50%, 98.39% ± 2.50%, and 99.44% ± 1.11% using the DLG model, while 71.38% ± 0.63%, 63.13% ± 2.87%, and 85.59% ± 6.66% using traditional GWAS. Similar results were obtained from the other two intergroup classifications.

**Conclusion:**

The DLG model can achieve higher accuracy and sensitivity when applied to progression of AD. More importantly, we discovered several novel genetic biomarkers of AD progression, including rs6311 and rs6313 in HTR2A, rs1354269 in NAV2, and rs690705 in RFC3. The roles of these novel loci in AD should be explored in future research.

## 1. Introduction

Alzheimer's disease (AD) is the most common type of dementia and is an irreversible, progressive neurological brain disorder typically beginning with mild memory decline; in time, it can seriously impair an individual's ability to carry out daily activities and lead to loss of autonomy [1, 2]. Mild cognitive impairment (MCI) is a preclinical stage of AD, in which individuals have no obvious cognitive behavioral symptoms but can show subtle prodromal signs of dementia [3, 4]. It is widely recognized that early detection of AD and MCI is essential to slowing progression.

Among factors that influence AD progression, common genetic variants are major risk factors [5]. Currently, the development of cheap comprehensive genetic testing of peripheral blood has brought dramatic changes to studies of the mechanisms of disease development. In recent decades, several genes have been associated with AD risk based on full-genome genotyping arrays using blood samples [6, 7]. For instance, genomics analysis showed APOE to be the most strongly associated AD risk gene [8]. In addition, the CLU, PICALM, SORL1, BIN1, and TOMM40 genes have also been identified as AD risk factors in the literature [7, 9, 10].

Technological advances [11] have allowed analysis of millions of nucleotide polymorphisms from thousands of subjects, including advanced genome-wide association studies (GWAS) and whole genome sequencing [12–16] that have increased our understanding of the genetic complexity of AD susceptibility. For instance, recent GWAS from the Alzheimer's Disease Neuroimaging Initiative (ADNI) have related known AD risk genes to differences in rates of brain atrophy and biomarkers of AD in the cerebrospinal fluid [17]. Moreover, the International Genomics of Alzheimer's Project studied 74046 participants, confirming nearly all of the previous genetic risk factors and identifying 12 new susceptibility loci for AD [18]. Therefore, genomics analysis, especially GWAS analysis, has yielded important advances in AD research.

However, there are some limitations of GWAS. Firstly, traditional GWAS intergroup analysis is distorted by differences in sample sizes [19]. Secondly, traditional GWAS analysis is strongly dependent on prior knowledge and hand coding, which requires much time and energy and risks bias or errors in data entry [16] that can result in poor repeatability. Moreover, although traditional GWAS analysis can assure high specificity of disease screening, accuracy, and sensitivity are relatively low. In practice, false positives are preferred over false negatives in order to avoid omissions in disease screening. Therefore, alternative analytical tools would help to drive novel hypotheses and models.

Deep-learning algorithms implemented via deep neural networks can automatically embed computational features to yield end-to-end models that facilitate discovery of relevant highly complex features [20]. Seminal studies in 2015 demonstrated the applicability of deep neural networks to DNA sequence data [21, 22]. Deep convolutional neural networks (CNNs) have been used in recent studies to predict various molecular phenotypes on the basis of DNA sequence alone. Applications include classifying transcription factor binding sites, predicting molecular phenotypes such as DNA methylation, microRNA targets, and gene expression [23–27]. In addition, CNNs have been utilized to call genetic variants [28] and classify genetic mutations in tumors [29]. Multitask and multimodal models and transfer learning have also been developed in genomics [30, 31]. In this work, we hypothesize that deep-learning genomics (DLG) can be applied to AD and outperform traditional GWAS analysis. We propose a DLG method to replace traditional GWAS analysis for multitasking classification of AD progression and use this approach to seek novel genetic biomarkers of AD susceptibility.

## 2. Materials and Methods

**Figure 1:**
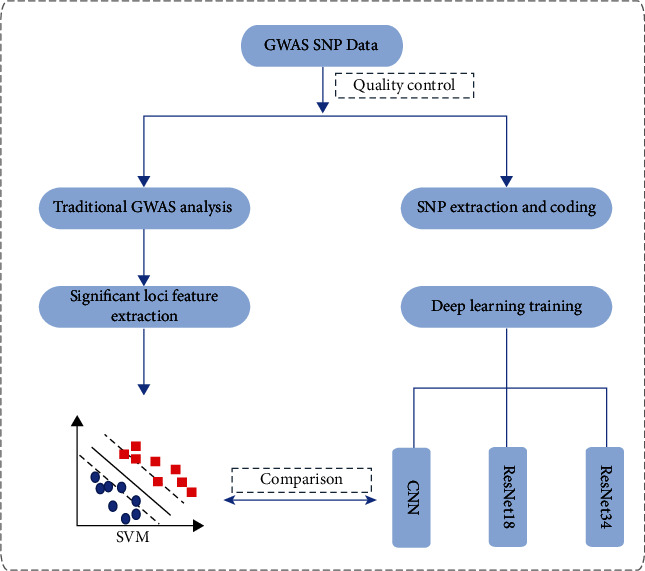
The flowchart of experimental procedures in this study.

The experimental workflow of this study consisted of three steps as shown in Figure 1. First, we conducted quality control and SNP genotype coding for SNP genotype data. Second, we used the deep residual network ResNet for DLG. The goal of the deep residual network was to obtain a model by supervised learning for prediction and extraction of DLG features. The details of this process are described in detail in the following sections. Finally, we investigated interpretability of the DLG model by applying Gradient-weighted Class Activation Mapping (Grad-CAM).

### 2.1. Subjects

Data used in the preparation of this study was obtained from the ADNI database (http://adni.loni.usc.edu/). ADNI was launched in 2004 by the National Institute on Aging, the National Institute of Biomedical Imaging and Bioengineering, the Food and Drug Administration, private pharmaceutical companies, and nonprofit organizations, as a $60 million, 5-year public-private partnership. In this study, 1461 individuals (622 AD, 473 MCI, and 366 healthy controls (HCs)) from the ADNI 1, ADNI 2, and ADNI GO cohorts of the ADNI database were included. Meanwhile, the following data from the 1461 ADNI participants was downloaded: Illumina SNP genotyping data, demographic information, and diagnosis information. Written informed consent was obtained from all participants, and the study was conducted with prior institutional review board approval. Clinical characteristics, including age, sex, education, and Montreal Cognitive Assessment (MoCA) results, were collected and are listed in Table 1.

The subjects were of age 55-90 (inclusive) years. The detailed ADNI eligibility criteria are available from http://adni.loni.usc.edu/methods/documents/. In brief, eligibility criteria for these participants were as follows: (1) normal subjects: a Clinical Dementia Rating (CDR) of 0, nondepressed, non-MCI, and nondemented; (2) MCI subjects: a memory complaint, objective memory loss measured by education adjusted scores on Wechsler Memory Scale 7/Logical Memory II, a CDR of 0.5, absence of significant levels of impairment in other cognitive domains, essentially preserved activities of daily living, and an absence of dementia; (3) AD: CDR of 0.5 or 1.0 and met the National Institute of Neurological and Communicative Disorders and Stroke and Alzheimer's disease and Related Disorders Association criteria for probable AD [32]. Specific psychoactive medications were excluded.

We investigated two groups of subjects using SNP genotype data collected from the ADNI databases. Our training and validation group contained 560 subjects with AD, 426 subjects with MCI, and 330 HC subjects. We used the SNP genotype data from this group to establish and test the validity of our predictive models. Our test group consisted of 62 AD subjects, 47 MCI subjects, and 36 HC controls, and we used the SNP genotype data to evaluate the diagnostic value of the predictive models.

### 2.2. DNA Isolation and SNP Genotyping

SNP genotyping for more than 620,000 target SNPs was completed on all ADNI participants using the following protocol. First, a total of 7 mL of blood was taken from each participant and stored in EDTA-containing Vacutainer tubes, and genomic DNA was extracted using the QIAamp DNA Blood Maxi Kit following the manufacturer's protocol. Second, lymphoblastoid cell lines were established by transforming B lymphocytes with Epstein-Barr virus [33]. Fourteen genomic DNA samples were analyzed using the Human 610-Quad BeadChip according to the manufacturer's protocols. Before starting the assay, a 50 ng sample of genomic DNA from each participant was examined qualitatively on a 1% Tris-acetate-EDTA agarose gel to check for degradation. Degraded DNA samples were excluded from further analysis. Third, samples were quantitated in triplicate with PicoGreen® reagent and diluted to 50 ng/L in TrisEDTA buffer (10 mM Tris, 1 mM EDTA, pH 8.0). A total of 200 ng of DNA was denatured, neutralized, and amplified for 22 hours at 37°C, and then fragmented with FMS reagent (Illumina) at 37°C for 1 hour, precipitated with 2-propanol, and incubated at 4°C for 30 minutes. Fourth, the resulting blue precipitate was resuspended in RA1 reagent (Illumina) at 48°C for 1 hour. Samples were then denatured (95°C for 20 minutes) and immediately hybridized onto BeadChips at 48°C for 20 hours. The BeadChips were washed and subjected to single base extension and staining. Finally, the BeadChips were coated with XC4 reagent (Illumina), desiccated, and imaged on a BeadArray Reader (Illumina). Illumina BeadStudio 3.2 software was used to generate SNP genotypes from bead intensity data.

### 2.3. Quality Control and APOE Genotype

The following quality control (QC) steps were performed on the 1461 samples using PLINK v1.07 software. QC processes were conducted separately between the AD and HC groups, the HC and MCI groups, and the AD and MCI groups. SNPs and participants were excluded from the analysis if they failed to meet any of the following criteria [34]: call rate per SNP ≥ 90%; call rate per participant ≥ 90%; gender check; minor allele frequency (MAF) ≥ 5%; Hardy–Weinberg equilibrium test of *p* ≤ 10^−6^; PI_HAT < 0.5. After the QC procedure, the numbers of features considered for future analysis of each subject in the paired groups were as follows: 301,388 in the HC and MCI groups, 301,853 in the HC and MCI groups, and 301,138 in the MCI and AD groups. The overall genotyping rate for the remaining dataset was over 99.5%.

In addition, although the APOE gene is an important target gene in AD research, it was not available for all identified APOE SNPs on the Illumina array. Therefore, based on the reported APOE *ε*2/*ε*3/*ε*4 status, the genotypes of the unavailable APOE SNPs were added manually to ADNI genotype data before assessing sample quality.

### 2.4. SNP Genotype Coding

A single-nucleotide polymorphism is a DNA sequence variation which occurs when a single nucleotide (A, T, C, or G) in the genome differs among members of a biological species or across paired chromosomes. Based on the satisfactory ADNI GWAS SNP data of this study, we encoded SNPs using the following coding scheme: 1 refers to A, 2 refers to T, 3 refers to C, and 4 refers to G.

### 2.5. GWAS Analysis

In the multitasking classification of this study, GWAS analysis, which has emerged as a popular tool for identifying genetic variants associated with disease risk, was designed to be compared with deep-learning models. Standard analysis of a case-control GWAS involves assessing the association between each individual genotyped SNP and disease risk. Manhattan and quantile–quantile (*Q*–*Q*) plots were used to visualize the GWAS results. All association results surviving the significance threshold of *p* < 1.66*e*^−7^ were saved and prepared for subsequent pattern analysis.

### 2.6. Deep-Learning Genomics Model Based on ResNet

The DLG model acted as a feature encoder, which had a significant impact on classification. In this study, we applied ResNet, a deep residual network, to the classification between AD and HC groups, AD and MCI groups, and HC and MCI groups. Residual units were added to the deep residual network on the basis of CNNs.

A CNN, the most effective type deep-learning model, is generally composed of three types of layers: convolutional, pooling, and fully connected. The following describes the operation of a CNN. The first step is to convolve the input sequences with a set of filter kernels; all the features active at different positions after convolution constitute the feature map [35]. A nonlinear activation function, typically a rectified linear unit (ReLU), is applied on each layer and on the sum of the feature maps. The operation of the convolutional layer and ReLU can be expressed as follows:

(1) $$\begin{array}{c} C_{n}^{r}=\text{ReLU}\left(\underset{m}{\sum }v_{m}^{r-1}*w_{n}^{r}+b_{n}^{r}\right),\\ \left\{\begin{array}{c} \text{ReLU}\left(y_{n}\right)=\max\left(0,y_{n}\right),\\ y_{n}=\underset{m}{\sum }v_{m}^{r-1}*w_{n}^{r}+b_{n}^{r}, \end{array}\right. \end{array}$$

where *C*_*n*_^*r*^ is the *n*^th^ output of the *r*^th^ convolutional layer, *n* represents the number of filters in the *r*^th^ layer, *w*_*n*_^*r*^ and *b*_*n*_^*r*^ are, respectively, the weight and bias of the *n*^th^ filter of the *r*^th^ layer, *v*_*m*_^*r*−1^ is the *m*^th^ output of previous layer *r* − 1, and ^∗^ denotes the convolutional operation.

Next, the resulting feature map is processed through the pooling layer by taking either the mean or maximum activation over disjoint regions for each channel [20, 35]. By sequential combination of convolutional and pooling layers, a multilayer structure is built for feature description. Lastly, the fully connected layers are employed for classification. In total, when given a training set {*X*_*j*_}_*j*_, the learning process of a CNN with *K* convolutional layers, whose filter parameters are {*W*_*i*_}_*i*=1_^*K*^, the bias values are {*b*_*i*_}_*i*=1_^*K*^, and *D* refers to classification layers, can be represented as an optimization learning task:

(2) $$\begin{array}{c} \underset{{\left\{W_{i}\right\}}_{i=1}^{K},{\left\{b_{i}\right\}}_{i=1}^{K}}{\min}\underset{j}{\sum }L\left(h\left(X_{j}\right),f\left({\left\{W_{i}\right\}}_{i=1}^{K},{\left\{b_{i}\right\}}_{i=1}^{K},D\right)\right), \end{array}$$

where *L* is the loss function that represents the cost difference between the true label *h*(*X*) and the predictive label from the CNN model *f*(*X*, {*W*_*i*_}_*i*=1_^*K*^, {*b*_*i*_}_*i*=1_^*K*^, *D*).

Based on the CNN model, the greatest advantage of the ResNet framework lies in adding identity mapping that is performed by the shortcut connections, the outputs of which are added to the outputs of the stacked layers [36]. Therefore, the ResNet addressed the degradation problem and added neither extra parameters nor computational complexity. The formula for residual learning was designed as follows: the desired underlying mapping is denoted as *H*(*x*), and the stacked nonlinear layers were allowed to fit a separate mapping of *ϝ*(*x*, Θ) = *H*(*x*) − *x*. The original mapping was recast into *F*(*x*, Θ) + *x*. Thus, the overall representation of the residual block was as follows:

(3) $$\begin{array}{c} H\left(x\right)=ϝ\left(x,\Theta \right)+x. \end{array}$$

The formulation of *ϝ*(*x*, Θ) + *x* can be realized by feedforward neural networks using “shortcut connections.” A deep residual network can be established by stacking a series of residual blocks. Specifically, there were two steps in the process: forward computation and backward propagation. When *K* residual blocks are chosen to stack, the forward propagation of such a structure can be expressed by

(4) $$\begin{array}{c} x_{K}=x_{0}+\overset{K}{\underset{r=1}{\sum }}ϝ\left(x_{r-1},\Theta_{r-1}\right), \end{array}$$

where *x*_0_ and *x*_1_ are the input and the output of the residual network, respectively, and Θ_*r*_ = {*θ*_*r*,*l*_|_1≤*l*≤*L*_} is the weight related to the *r*_th_ residual block, *L* being the number of layers within the block.

Likewise, the back propagation of the overall loss of the neural network to *x*_0_ can be denoted as

(5) $$\begin{array}{c} \frac{\partial L}{\partial x_{0}}=\frac{\partial L}{\partial x_{K}}\left(1+\frac{\partial }{\partial x_{0}}\overset{K}{\underset{i=1}{\sum }}ϝ\left(x_{i-1},\Theta_{i-1}\right)\right), \end{array}$$

where *L* is the whole loss function of the neural network.

Before modeling using the above procedures, each subject's SNP genotype data was cropped after quality control and mapped to 776 × 776 pixels. The pathology type was encoded to one-hot, which was the label. Thereafter, in the training stage, SNP genotype data was fed into the network to update model parameters via backward propagation with the Adam algorithm, a first-order gradient-based optimization algorithm which has been proven to be computationally efficient and appropriate for training deep neural networks. The outputs of the network were used as the classification results, and the crossentropy of the outputs was calculated as the loss function. More specifically, the output of the network for each individual SNP could be a binary value. 1 represented the highest probability of being AD subjects, while 0 represented highest probability of being HC subjects.

We adopted ResNet18 and ResNet34 frameworks in this study. Meanwhile, we also utilized a traditional CNN model for the comparative experiments of classification. In the ResNet models, we set learning rate into 1*e*^−3^ and applied the Adam optimizer to update the model parameters with the batch size of 8. The maximum number of iterations was set into 20. Note that we used L2 regularization in this step to prevent the overfit of our model. For adjusting the CNN model parameters, we set learning rate into 1*e*^−2^ and applied the Adam optimizer to update the model parameters with the batch size of 8. The maximum number of iterations was set to 30. Above deep-learning models were processed on a GPU (graphics processing unit, GTX 1080 Ti acceleration of PyCharm 3.5).

For investigating the interpretability of the DLG model, the last convolutional layer of the last res-block was made transparent in order to extract DLG features by applying Grad-CAM and two-sample *t*-tests. For the first step, the last convolutional layer of the last res-block was chosen to extract normalized DLG features. Subsequently, using a two-sample *t*-test with a false discovery rate [37, 38], we compared the *Z*-coefficients of the AD and HC groups, the HC and MCI groups, and the MCI and AD groups.

### 2.7. Classification

In this study, the subjects of multitasking classification were randomly divided into one training group and one independent test group at a ratio of 9 : 1 as shown in Table 1. The training group was then used to optimize the model parameters. We also randomly chose 25% of training group to form a validation group to guide the choice of hyperparameters.

On the one hand, we conducted training of several deep-learning models, including ResNet18, ResNet34, and a traditional CNN, and compared classification performance in order to screen for the optimum DLG. On the other hand, in order to verify the diagnostic capabilities of the DLG model compared with traditional GWAS analysis, we also designed comparative trials. Among all the gene indicators, theta proved to be the most directly related to SNP changes. APOE *ε*4 status and the normalized theta-value of the significant SNP loci found in this study were seen to be genetic predictors, and we used the support vector machine (SVM) with the linear kernel 500 times for classification of traditional GWAS.

To evaluate classification performance, we repeatedly conducted 5-fold crossvalidation in the training group. Accuracy, sensitivity, and specificity were used to evaluate the results. The mathematical expression of the three parameters was as follows:

(6) $$\begin{array}{c} Accuracy=\frac{Tn+Tp}{Tn+Tp+Fn+Fp},\\ Sensitivity=\frac{Tp}{Tp+Fn},\\ Specificity=\frac{Tn}{Tn+Fp}, \end{array}$$

where *Tn*, *Tp*, *Fn*, and *Fp* denote, respectively, true negatives, true positives, false negatives, and false positives.

A receiver-operating characteristic (ROC) curve was produced to intuitively compare the results of the different approaches, and the area under the curve (AUC) of the ROC was computed to quantitatively evaluate classification performance.

### 2.8. Statistical Analysis

Demographic characteristics were compared between groups using a two-sample *t*-test or the chi-square test. In addition, a two-sample *t*-test of the extracted features was applied as a criterion to estimate the differences in DLG features between AD patients and HCs, AD patients and MCIs, and HCs and MCIs. All statistical analyses were performed using SPSS Version 22.0 software (SPSS Inc., Chicago, IL) and Matlab2016b (Mathworks Inc., Sherborn, MA, United States). All *p* values < 0.05 were considered significant.

## 3. Results

### 3.1. Outcomes of GWAS Analysis

**Figure 2:**
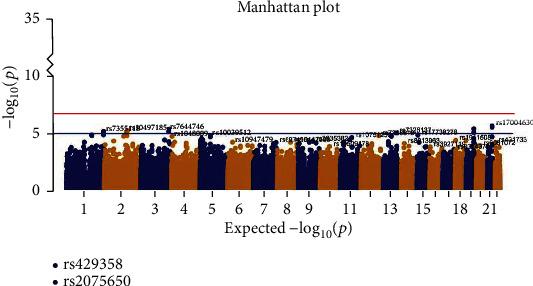
Manhattan plot of genome-wide association study (GWAS) between AD and HC groups. The *y*-axis shows the *p* value (on the –log10 scale) for each association test. The *x*-axis is the chromosomal position of each SNP. The horizontal lines in the Manhattan plot display the cutoffs for two significant levels: blue line for *p* < 10^−5^ (generally significant level) and red line for *p* < 1.66*e*^−7^.

**Figure 3:**
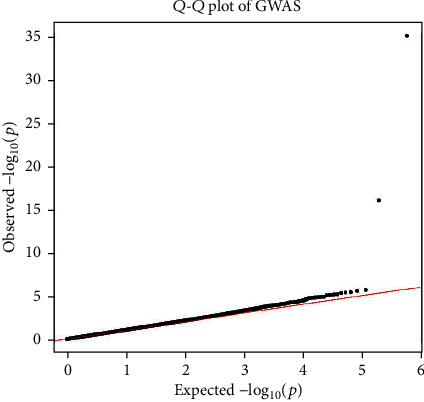
*Q*–*Q* plot of genome-wide association study (GWAS) between AD and HC groups. Genomic inflation factor is 1.084.

We carried out case-control GWAS analysis between the AD and HC groups and observed two genome-wide significant loci on chromosome 19, including rs429358 (APOE, the epsilon 4 marker) and rs2075650 (TOMM40). Figures 2 and 3 show the resulting Manhattan and *Q*–*Q* plots, and Table 2 summaries the SNPs that achieved genome-wide significance. The *p* value used to assess significant differences was calculated as *p* = 0.05/*N*, where *N* indicates the number of satisfied SNPs.

### 3.2. Classification Performance

**Figure 4:**
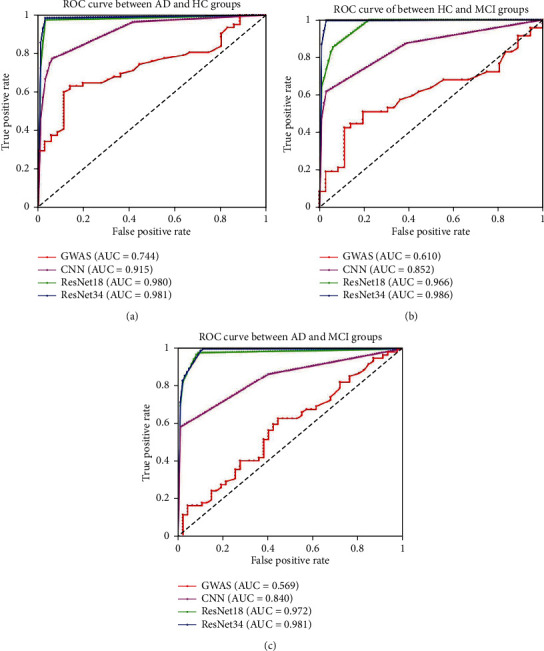
ROC curve of the performance of different classification approaches in multitasking classification. (a) ROC curve between AD and HC groups. (b) ROC curve between MCI and HC groups. (c) ROC curve between AD and MCI groups.

Table 3 lists the performance of the different multitasking classification methods, including classification accuracy, sensitivity, specificity, and AUC. Taking the result of classification between the AD and HC test group subjects as an example, accuracy, sensitivity, specificity, and AUC were, respectively, 71.38% ± 0.63%, 63.13% ± 2.87%, 85.59% ± 6.66%, and 0.744, for the GWAS analysis, 92.45% ± 8.13%, 93.87 ± 12.26, 90.00 ± 15.97, and 0.915 for the CNN model, 97.96 ± 1.71, 97.42 ± 3.16, 98.89 ± 1.36, and 0.980 for ResNet18, and 98.78% ± 1.50%, 98.39% ± 2.50%, 99.44% ± 1.11%, and 0.981 for ResNet34. We found that the deep-learning model exhibited high accuracy, sensitivity, and specificity, whereas accuracy and sensitivity were low for the GWAS analysis. Therefore, we concluded that deep-learning models were superior to traditional GWAS analysis for classification. And compared with the CNN model, the results using ResNet were more robust and stable. These results were the same using the other two group-level classifications. Based on these results, ResNet34 was chosen for the DLG model because the observed classification performance was optimal among the several deep-learning models. A more intuitive comparison is provided by the ROC curves of the multitasking classification shown in Figure 4.

### 3.3. Interpretability of the DLG Model

Setting a threshold of *p* < 0.05, more than ten thousand SNP loci showed differences between the groups, and even the significance of the most frequently identified loci was below 0.001.

Firstly, we compared the significant SNPs with those previously identified by GWAS as genetic susceptibility factors. Almost one hundred SNP loci between AD patients and HCs were consistent with findings from previous studies. Likewise, more than one hundred associated SNP loci were also found between the AD and MCI groups and between the HC and MCI groups.

**Figure 5:**
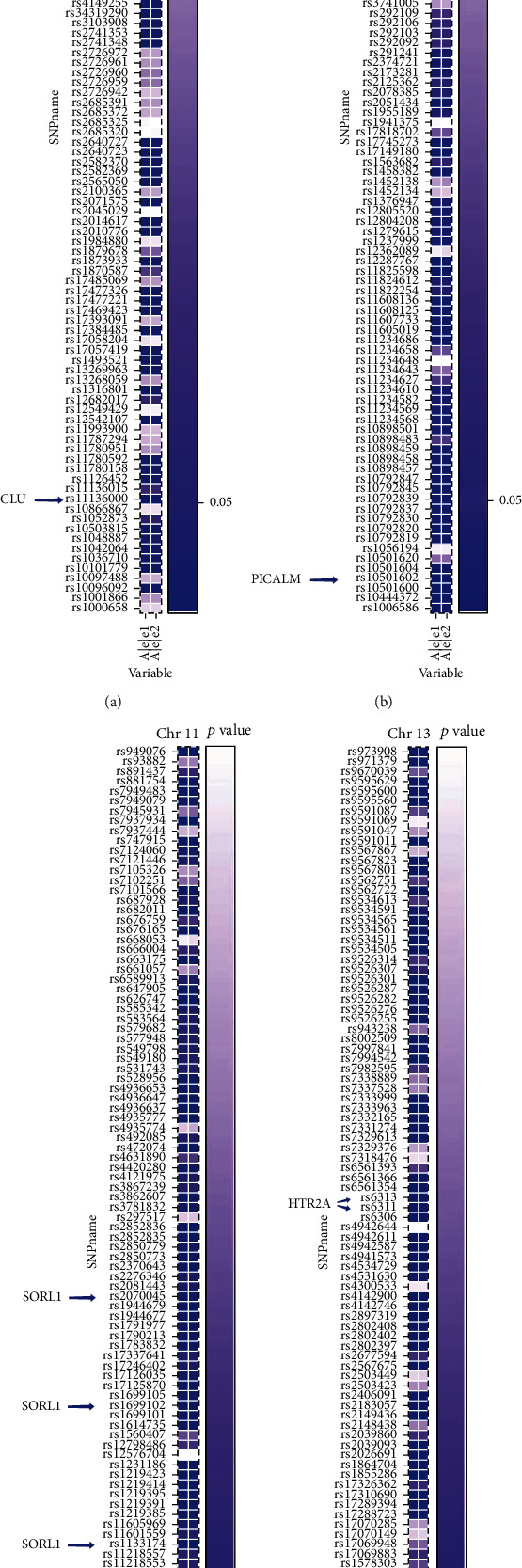
Visualization for part of shared significant SNPs in AD multitasking classification at *p* threshold of 0.05. Rs11136000 (CLU) on chromosome 8 (a); rs543293, rs10501602, and rs3851179 (PICALM) on chromosome 11 (b); rs2070045, rs1699102, and rs1133174 (SORL1) on chromosome 11 (c); and rs6311 and rs6313 (HTR2A) on chromosome 13 (d) are shown successively from left to right.

Secondly, we sought significant SNP loci among three classification tasks. The gene regions of sixty-six SNP loci were shared in different stages of AD progression.  Table 4 summarizes the sixty-six shared significant SNP loci among the three classifications, including, e.g., the well-known CLU, PICALM, and SORL1 gene regions. For rs11136000 (CLU) in chromosome 8, its *p* values were 6.63*e*^−4^ between the AD and HC groups, 8.37*e*^−6^ between the MCI and HC groups, and 1.49*e*^−7^ between MCI and AD groups. In addition, three SNP loci, rs543293, rs10501602, and rs3851179, were found in the PICALM gene region of chromosome 11, and the *p* values of rs3851179 for the comparisons of the three groups were 6.00*e*^−3^, 1.06*e*^−6^, and 1.51*e*^−20^, while the *p* values of rs543293 were 4.65*e*^−3^, 3.43*e*^−8^, and 2.27*e*^−13^, and the *p* values of rs3851179 were also much less than 0.001. These results are well supported by previous studies. Other significant results are detailed in Table 4, and the heatmaps of significant SNPs in chromosomes 8, 11, and 13 are shown in Figure 5. The horizontal axis represents major and minor alleles, and the vertical axis represents the *p* value of SNP loci in the chromosomes. We observed some distinct differences, for example, between rs11136000, rs3851179, and surrounding loci.

In addition, except for those in Table 4, there were also several SNP loci showing an association with AD progression in their respective classifications. Several also have been reported and confirmed in previous large-scale GWAS studies, including APOE, BIN1, CHRM1, and TOMM40 with *p* values much less than 0.001. Furthermore, it is notable that rs6311 and rs6313 in the HTR2A gene region, rs1354269 in the NAV2 gene, and rs690705 in the RFC3 gene all exhibited significant differences among the three classifications. For instance, the *p* values of rs6311 were 1.96*e*^−5^, 2.52*e*^−3^, and 1.48*e*^−11^ between the respective groups, and the *p* values of rs6313 were 3.21*e*^−5^, 4.55*e*^−3^, and 2.05*e*^−12^. An understanding of the roles of these novel loci in AD requires future study.

All of the information above was deposited in the DisGeNET database (http://www.disgenet.org/home/), a discovery platform containing one of the largest publicly available collections of genes and variants associated with human disease.

## 4. Discussion

This study used a comparison of the performance of several different deep-learning models as a basis for proposing a deep-learning genomics method based on ResNet34. The classification results indicate that the DLG model offers a higher diagnostic value than traditional GWAS analysis.

### 4.1. Outcomes of GWAS Analysis

In GWAS analyses, two SNPs have been identified at the *p* < 1.66*e*^−7^ significance level: APOE SNP rs429358 was determined to be the most significant genetic risk factor for AD. And the second most significant factor, TOMM40 SNP rs2075650, was found to be adjacent to the APOE SNP [10]. These results are consistent with previous studies. Although these SNP loci were identified by GWAS, traditional GWAS analysis suffers from being influenced by small sample size. Because other common genetic risk factors may have a much smaller impact on risk than the APOE gene, novel risk factors present in small samples may go undetected by GWAS analysis. Several previous studies have also demonstrated an explicit relationship between sample size and the number of significant differences in traits identified by genome-wide association studies [18, 19].

### 4.2. Classification Performance

In this study, in order to construct a deep-learning genomics model, we compared the performance of several deep-learning classification methods, including a simple CNN model, ResNet18, and ResNet34. As shown in Table 3, we observed that the results of the deep residual network were superior to those of a simple CNN, and in the process of training the model, the ResNet models exhibited robustness and stability superior to those of CNNs, and furthermore, ResNet34 was superior to RseNet18. Therefore, we chose ResNet34 as the final DLG model. More importantly, we compared the performance of the DLG model and traditional GWAS analysis under the same conditions and found the classification results of the DLG model to be superior. These results suggest that the deep-learning algorithm is effective in genome applications and that development of deep-learning genomics is worthy of further exploration.

### 4.3. Interpretability of the DLG Model

When we interpreted the DLG model, we found more than one thousand SNP loci with significant differences between AD patients and HCs, between the MCI and AD groups, and between the HC and MCI subjects. As is well known, rs11136000 (CLU), rs3851179 (PICALM), rs2070045 (SORL1), and rs1699102 (SORL1) have previously been identified as risk factors for AD [7, 9, 39]. Notably, they were all included among the sixty-six significant SNP loci shared in the three classification tasks in this study (as shown in Table 4 and Figure 5). For example, previous studies have shown that CLU modulates A*β* metabolism and is involved in A*β* clearance or acts as a chaperon for protein degradation [40]. PICALM, as an adaptor protein involved in clathrin-mediated endocytosis, regulates amyloid precursor protein (APP) internalization and subsequent A*β* generation, contributing to brain amyloid plaque load via its effect on A*β* metabolism [41, 42]. In addition to the analysis of the above identical SNP loci found among the three classification tasks, several differential loci were identified among one or two classification tasks, which are also consistent with previous research. Rs10194375 (BIN1), a protein that may be associated with tau-mediated pathology was identified as being significant between the AD and HC groups and the AD and MCI groups. In addition, rs2075650 (TOMM40), rs405509 (APOE), and rs429358 (APOE) were identified as significant between the HC and MCI groups and the MCI and AD groups. In summary, the DLG model is able to identify differential genomics in multitasking classification.

Most importantly, in addition to those shown to associate with AD in the past, we found several new SNP loci, including rs6311 (HTR2A), rs6313 (HTR2A), rs1354269 (NAV2), rs1946518 (IL18), rs1799986 (LRP1), rs690705 (RFC3), and rs7943454 (LUZP2), whose *p* values were highly significant(as shown in Table 4). Rs6311 and rs6313 are in the HTR2A gene region. The HTR2A gene in humans is located on chromosome 13 and consists of exons separated by only two introns and encodes one of the receptors for serotonin. According to previous publications, HTR2A has received much attention in many psychiatric disorders such as mood disorders, attention deficit hyperactivity disorder, anxiety disorders, and schizophrenia. On the one hand, some studies have shown that medications for mood disorders and related conditions work by blocking 5-HT2A and altering the function of certain brain circuits. And blocking 5-HTR2A also seems to improve the effects of some antidepressants [43]. On the other hand, the numbers of the postsynaptic receptor HTR2A are reduced in the neocortex, and it seems to be involved in memory via its role in cortical pyramidal cells. For example, in AD research, HTR2A receptor densities in the brains of AD subjects were found to be reduced compared with age-matched controls, and the researchers also found this reduction correlated with the rate of decline of cognitive scores [44]. Hence, since subjects with AD or mild cognitive impairment exhibit depression and anxiety to various degrees, it is worth exploring whether rs6311 and rs6313 of the HTR2A gene contribute to AD susceptibility. Another significant locus identified here was rs1354269 located in the NAV2 gene region. The NAV2 gene, which encodes a member of the neuron navigator gene family, is highly expressed in brain and is involved in the development of the nervous system. Hence, the role of the NAV2 gene in AD is also worthy of future investigation. In addition, rs690705 of the RFC3 gene region also exhibited a significant difference in group-level classifications, and its impact on AD should be examined in the future.

## 5. Limitations

It is worth noting some limitations of this study. Firstly, only gene sequences were used as inputs to the DLG classification. In the future work, we plan to combine gene sequences with clinical data and brain imaging [45] to facilitate investigation of the mechanisms of AD progression by deep-learning genomics and deep-learning radiomics approaches. Secondly, we only classified information from the ADNI dataset in this study, so the results could be strengthened by including other datasets such as the Chinese populations. Thirdly, the number of subjects represented in this study may be limiting. Lastly, although this study has demonstrated the feasibility of DLG approach, it will be important to further explore the interpretability of deep-learning genomics.

## 6. Conclusions

In conclusion, the current study suggests that the deep-learning genomics approach is effective for multitasking classification research on AD progression and outperforms traditional GWAS analysis. Moreover, the several novel SNP loci identified in the DLG approach including rs6311 and rs6313 in HTR2A, rs1354269 in NAV2, and rs690705 in RFC3 are worthy of further exploration to better understand the mechanisms of AD.

## Figures and Tables

**Table 1:** Clinical and baseline demographic characteristics of all participants.

Groups	Gender (M/F)	Age (years)	Education	MoCA
*Training and validation group(n* = 1316)				
AD (*n* = 560)	320/240^a^	74.42 ± 7.26	15.54 ± 2.85^a^	17.18 ± 5.05^a,b^
MCI (*n* = 426)	255/171^c^	73.27 ± 7.39	15.98 ± 2.78^c^	23.62 ± 2.95^b,c^
HC (*n* = 330)	163/167^a,c^	73.80 ± 5.84	16.46 ± 2.54^a,c^	25.88 ± 2.42^a,c^
*Test group(n* = 145)				
AD (*n* = 62)	41/21^a^	75.71 ± 7.99	15.55 ± 3.32	13.91 ± 6.82^b^
MCI (*n* = 47)	30/17^c^	75.56 ± 7.94	14.81 ± 3.70	22.75 ± 3.31^b^
HC (*n* = 36)	14/22^a,c^	75.45 ± 3.49	15.58 ± 3.59	—

Data of age and education were presented as mean ± standard deviation. MoCA: Montreal Cognitive Assessment. Group-level two-sample *t* test is conducted for age, education, and MoCA. Group-level chi-square test is conducted for gender. ^a^*p* value HC vs. AD; ^b^*p* value AD vs. MCI; ^c^*p* value HC vs. MCI.

**Table 2:** SNP summaries reaching genome-wide significance after GWAS.

SNP	Position	Chr	Region or closest gene	Major/minor alleles	*p* value	OR
rs429358	44908684	19	APOE	C/T	5.407*e*-36	4.348
rs2075650	50087459	19	TOMM40	G/A	7.19*e*-17	2.737

*p* < 1.66*e*^−7^ for SNPs listed above. Chr: chromosome.

**Table 3:** Performance of different classification approaches in multitasking classification.

Model	Accuracy (%)	Sensitivity (%)	Specificity (%)	AUC
*AD and HC groups*				
GWAS analysis	71.38 ± 0.63	63.13 ± 2.87	85.59 ± 6.66	0.744
CNN model	92.45 ± 8.13	93.87 ± 12.26	90.00 ± 15.97	0.915
ResNet18	97.96 ± 1.71	97.42 ± 3.16	98.89 ± 1.36	0.980
ResNet34	98.78 ± 1.50	98.39 ± 2.50	99.44 ± 1.11	0.981
*MCI and HC groups*				
GWAS analysis	56.99 ± 1.55	96.08 ± 13.92	5.94 ± 21.65	0.510
CNN model	87.47 ± 16.64	99.57 ± 0.85	71.67 ± 38.75	0.852
ResNet18	97.59 ± 3.73	100.00 ± 0.00	94.44 ± 8.61	0.966
ResNet34	99.52 ± 0.60	99.57 ± 0.85	99.44 ± 1.11	0.986
*AD and MCI groups*				
GWAS analysis	58.97 ± 0.00	72.18 ± 0.01	41.54 ± 0.01	0.569
CNN model	86.42 ± 16.02	97.42 ± 4.40	71.91 ± 39.21	0.840
ResNet18	97.80 ± 1.24	97.74 ± 2.41	97.87 ± 3.30	0.972
ResNet34	98.90 ± 1.78	100.00 ± 0.00	97.45 ± 4.13	0.981

The methods are conducted with crossvalidation, and their results are given as mean ± standard deviation.

**Table 4:** Shared significant SNPs in AD multitasking classification at *p* threshold of 0.05.

SNP loci	Chr	Position	Region or closest gene	Major/minor alleles	*p* value HC vs. AD	*p* value HC vs. MCI	*p* value AD vs. MCI
rs12091371	1	238671675	FMN2	A/G	0.002227833	0.001509792	2.27881*E*-09
rs12129547	1	238761878	GREM2	T/C	0.002767757	0.019531535	3.93396*E*-11
rs1801131	1	11777063	MTHFR	C/A	0.000154746	0.007333869	6.18756*E*-21
rs1801133	1	11778965	MTHFR	T/C	0.000192989	0.009566143	5.35043*E*-21
rs17034806	2	109002337	RANBP2	G/A	0.002645921	0.028425309	3.04207*E*-10
rs243034	2	60456396	MIR4432HG	G/A	0.038985718	0.009492742	1.40355*E*-08
rs4676049	2	109001689	RANBP2	T/C	0.002215778	0.034993277	4.51555*E*-10
rs6714710	2	97711518	ZAP70	G/T	0.038909198	0.000768783	7.036*E*-09
rs1498853	3	69691797	NAN^a^	G/A	0.000708166	0.012435495	6.43721*E*-09
rs2289506	3	101547592	NIT2	T/C	0.000412705	0.004914541	1.07429*E*-18
rs288496	3	69714739	NAN^a^	T/C	0.002172998	0.009409715	4.5892*E*-09
rs3864101	3	188862449	NAN^a^	T/G	0.038241862	0.022506051	6.17468*E*-08
rs989692	3	156284059	MME	T/C	0.00243336	0.016131413	5.91293*E*-09
rs3796529	4	57492171	REST	A/G	0.048696066	0.004826601	7.08294*E*-25
rs753129	4	56363188	NAN^a^	C/T	0.004469287	1.23015*E*-10	0.013855564
rs1925458	6	23486930	LOC102724749; LOC105374976	T/G	0.000567167	0.000409483	3.10404*E*-11
rs1980493	6	32471193	BTNL2; TSBP1-AS1	G/A	0.016858811	0.000751139	3.35304*E*-14
rs2651206	6	43321455	TTBK1	T/C	0.034368574	2.23746*E*-07	1.97506*E*-11
rs3734254	6	35502988	PPARD	C/T	0.001667544	0.000173601	2.21574*E*-11
rs3747742	6	41270496	TREML2	C/T	6.7184*E*-06	3.50103*E*-05	1.19671*E*-08
rs6455128	6	62755705	KHDRBS2	A/C	0.008767325	0.00013524	1.45915*E*-14
rs11767557	7	142819261	EPHA1-AS1	C/T	0.001139233	1.20661*E*-07	1.96401*E*-07
rs11771145	7	142820884	EPHA1-AS1	A/G	0.001112928	9.62693*E*-08	1.8557*E*-07
rs2227631	7	100556258	SERPINE1	G/A	0.003024669	8.25065*E*-08	7.24385*E*-07
rs6461569	7	21502301	SP4	C/T	0.025204688	1.41338*E*-06	0.001322394
rs6966915	7	12232513	TMEM106B	T/C	0.031810788	0.004328052	6.57464*E*-09
rs11136000	8	27520436	CLU	T/C	0.000663408	8.36729*E*-06	1.48921*E*-07
rs1975804	8	109360409	EIF3E; LOC105375704	C/T	0.000833268	0.003779274	0.040135088
rs1800977	9	106730271	ABCA1; LOC105376196	T/C	0.013227434	5.61278*E*-05	4.22923*E*-14
rs2007153	9	135493640	DBH	A/G	3.88584*E*-05	0.000993637	4.15766*E*-07
rs2066715	9	106627854	ABCA1	A/G	0.004157538	0.04271672	7.79217*E*-05
rs2283123	9	135505118	DBH	T/C	0.000105332	0.00221966	1.99182*E*-07
rs2740483	9	106730356	ABCA1; LOC105376196	C/G	0.009678488	6.53287*E*-05	3.81638*E*-14
rs4149313	9	106626574	ABCA1	G/A	0.003318514	0.040059489	7.22211*E*-05
rs4878104	9	89382811	DAPK1	T/C	0.002309724	6.11706*E*-05	1.46237*E*-14
rs4548513	10	67710331	CTNNA3	T/C	0.004692221	0.000992064	6.62732*E*-08
rs7070570	10	67534610	LOC105378340; CTNNA3	G/A	0.000655491	2.34147*E*-05	1.63609*E*-06
rs10501602	11	85359037	PICALM	G/A	0.004647595	3.42511*E*-08	2.27238*E*-13
rs1133174	11	121006965	SORL1	A/G	0.010544659	0.003255318	3.13279*E*-05
rs12805520	11	85308059	CCDC83	T/C	0.007162755	4.02484*E*-08	1.90295*E*-11
rs1354269	11	19330820	NAV2	C/T	0.010833795	6.42781*E*-08	2.65216*E*-24
rs1695	11	67109265	GSTP1	G/A	0.006309008	6.1645*E*-07	2.06528*E*-23
rs1699102	11	120962172	SORL1	C/T	0.013141699	0.001477832	6.19383*E*-05
rs17571	11	1739170	CTSD	T/C	0.013910348	1.48442*E*-08	1.58542*E*-20
rs1946518	11	111540668	IL18	T/G	0.006486007	1.8075*E*-06	7.21291*E*-22
rs2070045	11	120953300	SORL1	G/T	0.014545915	0.001140944	7.97485*E*-05
rs3851179	11	85546288	PICALM	A/G	0.00599555	1.06305*E*-06	1.50773*E*-20
rs543293	11	85497725	PICALM	A/G	0.004782682	1.26328*E*-07	7.85454*E*-18
rs6265	11	27636492	BDNF; BDNF-AS	A/G	0.009460455	1.96067*E*-07	1.42052*E*-28
rs7120118	11	47242866	NR1H3	C/T	0.034010947	0.011720707	5.90652*E*-22
rs7943454	11	24478242	LUZP2	T/C	0.01777448	1.41141*E*-06	2.00064*E*-30
rs1799986	12	55821533	LRP1	T/C	0.000875404	8.1991*E*-06	0.000948497
rs6311	13	46369479	HTR2A	T/C	1.95644*E*-05	0.002519461	1.48261*E*-11
rs6313	13	46367941	HTR2A	T/C	3.21121*E*-05	0.004551786	2.05426*E*-12
rs690705	13	33552918	RFC3	G/A	9.15016*E*-07	0.000123939	0.032894005
rs7989332	13	19948575	CRYL1	A/C	0.001947338	0.038324875	0.017831896
rs10137185	14	63845529	ESR2	T/C	0.000105484	0.00099597	2.08051*E*-22
rs1065778	15	49307498	MIR4713HG; CYP19A1	A/G	7.88465*E*-05	1.51664*E*-05	2.28151*E*-18
rs2278317	15	31848032	RYR3	G/A	0.019133823	5.18518*E*-05	4.11594*E*-07
rs3751592	15	49393870	CYP19A1; MIR7973-1; MIR7973-2	G/A	0.000333452	8.25536*E*-05	5.77922*E*-13
rs11075996	16	52415525	FTO	T/C	0.021538201	9.92354*E*-07	0.002856803
rs11075997	16	52416413	FTO	T/C	0.023256237	1.14854*E*-06	0.002461173
rs6499640	16	52327178	FTO	G/A	0.016928027	4.22569*E*-07	0.009148135
rs1050565	17	25600202	BLMH	G/A	0.045066195	6.1136*E*-06	1.19122*E*-06
rs391300	17	2163008	SRR	A/G	0.028223591	1.90848*E*-06	0.000379785
rs7946	17	17350285	PEMT	C/T	0.044709718	0.000412862	2.75631*E*-18

SNP: single-nucleotide polymorphism. Chr: chromosome. NAN^a^ represents uncertain gene of the SNP loci. *p* value (HC vs. AD) represents the difference value of each SNP loci between AD and HC groups. All *p* value are calculated by a two-sample *t* test for false discovery rate correction.

## Data Availability

The datasets presented in this study were obtained from ADNI (http://adni.loni.usc.edu/).
